# Knee extension range of motion and self-report physical function in total knee arthroplasty: mediating effects of knee extensor strength

**DOI:** 10.1186/1471-2474-14-33

**Published:** 2013-01-18

**Authors:** Yong-Hao Pua, Peck-Hoon Ong, Hwei-Chi Chong, William Yeo, Celia Tan, Ngai-Nung Lo

**Affiliations:** 1 Department of Physiotherapy, Singapore General Hospital, Singapore, Singapore; 2 Orthopaedic Diagnostic Centre, Singapore General Hospital, Singapore, Singapore; 3 Allied Health Division, Singhealth, Singapore, Singapore; 4 Department of Orthopaedic Surgery, Singapore General Hospital, Singapore, Singapore

## Abstract

**Background:**

Knee extensor strength and knee extension range of motion (ROM) are important predictors of physical function in patients with a total knee arthroplasty (TKA). However, the relationship between the two knee measures remains unclear. The purpose of this study was to examine whether changes in knee extensor strength mediate the association between changes in knee extension ROM and self-report physical function.

**Methods:**

Data from 441 patients with a TKA were collected preoperatively and 6 months postoperatively. Self-report measure of physical function was assessed by the Short Form 36 (SF-36) questionnaire. Knee extensor strength was measured by handheld dynamometry and knee extension ROM by goniometry. A bootstrapped cross product of coefficients approach was used to evaluate mediation effects.

**Results:**

Mediation analyses, adjusted for clinicodemographic measures, revealed that the association between changes in knee extension ROM and SF-36 physical function was mediated by changes in knee extensor strength.

**Conclusions:**

In patients with TKA, knee extensor strength mediated the influence of knee extension ROM on physical function. These results suggest that interventions to improve the range of knee extension may be useful in improving knee extensor performance.

## Background

Knee osteoarthritis (OA) is associated with substantial functional limitations in older adults [1]. Specifically, knee OA, along with hip OA, accounted for the largest proportion of disability in walking and stair climbing more than any other chronic diseases [2]. In patients with advanced stages of painful knee OA, although a total knee arthroplasty (TKA) can effectively restore function, 1 in 6 patients with TKA continue to have substantial physical function limitations [3]. Clearly, an in-depth understanding of the modifiable predictors of physical function and their aetiological pathways is needed which, in turn, could assist the refinement of interventions to improve function.

Clinically, two common knee impairments before and following a TKA are reduced knee extensor strength and deficits in knee extension range-of-motion (ROM) [4]. Although most [5-12] but not all [13] previous studies have implicated knee strength and knee extension ROM as important predictors of physical function, notably absent from these studies was an evaluation of the interrelation between knee extensor strength and knee extension ROM: previous studies – by their choice of standard regression analyses – assumed that these 2 knee measures have distinct and unrelated etiologies and that they act through different pathways to influence physical function.

Is it possible that knee extensor strength and knee extension ROM are interrelated? Based on what is known about the length-dependent nature of muscle force production, [14,15] we believe it is biologically plausible that knee extensor strength may play a role in the mechanistic pathway connecting knee extension ROM and physical function. Indeed, many activities of daily living – for example, level walking [16] – require the knee extensors to produce forces at lesser degrees (~30°) of knee flexion. Ostensibly, at a given knee joint angle near full extension, a flexion contracture may limit force production because the knee extensors – the monoarticular vastii in particular – are operating at a disadvantaged (lower) position on the ascending limb of their force-angle curves [14,15]. In as much as this concept may make sense and lead to the refinement of theoretical and intervention models, supporting data in patients with TKA are surprisingly sparse.

Because the interrelation between knee extensor strength and knee extension ROM is possible and important to understand clinically, we initiated the present study to examine whether changes in knee extensor strength mediate the association between changes in knee extension ROM and self-report physical function in a large group of patients before and following a TKA.

## Methods

### Settings and participants

Our study used a longitudinal pre- and post-TKA design that took advantage of the substantial differences in change for knee impairments and self-report physical function. The study involved 836 consecutive patients aged 50 years or older who underwent a primary TKA for knee OA performed by three high-volume surgeons at Singapore General Hospital, Singapore, from 3 January 2006 to 29 January 2009. To avoid potential confounding effects from a contralateral TKA operation, we excluded patients if they had a contralateral TKA within the 12 months before or 6 months after their index TKA (*n* = 152). We also excluded patients who (i) had a history of stroke or other neurological disorders (*n* = 30), (ii) had a history of lower limb fracture (*n* = 12), (iii) had previously undergone a hip arthroplasty or high tibial osteotomy (*n* = 26), (iv) had previously undergone a unicompartmental knee arthroplasty (UKA) on their index knee (*n* = 4), or (v) developed medical or surgical complications prior to the follow up session (*n* = 64). Finally, because this exploratory study is concerned with the effects of knee flexion contractures, we excluded patients with missing knee data (*n* = 48) and patients with knee hyperextension (extension ROM <0°) at the preoperative or follow-up assessment (*n* = 59). (The recruitment process is summarized in a flowchart in Additional file 1.) Thus, the sample for analysis comprised the remaining 441 patients who underwent a preoperative evaluation within 5 weeks prior to their operation. Of note, this sample size was not based on a formal power calculation but on all eligible patients in our database. Follow-up assessment was conducted approximately 6 months after the operation. All data were collected, as part of the clinical process, by physical therapists and entered into an electronic database per routine practice policies of our institution. All patients in this study were managed using a coordinated clinical pathway to ensure standardized medical, pharmacological, and rehabilitation care. Within two weeks post TKA, these patients began a 4- to 6-week rehabilitation program at the Singapore General Hospital outpatient physiotherapy clinic. The Centralized Institutional Review Board of Singhealth (CIRB), Singapore, approved the study and waived the need for informed consent due to the retrospective and anonymous nature of the study.

### Demographic variables

The demographic information collected in this study included age (in years), sex, ethnicity (Singaporean Chinese, as compared with others), and education (1 = primary school or less, 2 = vocational or secondary school, 3 = college or university). Data about comorbidities were retrieved from patients’ medical records by a research assistant using a checklist modeled after the Self-Administered Comorbidity Questionnaire [17]. Patients’ height and weight were also obtained and body mass index (BMI) was calculated as the ratio of weight to squared height (kg/m^2^).

### Self report physical function

At each assessment, patients were interviewed in either English or Mandarin using the Short Form-36 [18], of which we used the physical function subscale as the outcome variable and the bodily pain and mental health subscales as the study covariates. All subscales range from 0–100, with higher scores representing better health state. The English and Chinese versions of the SF-36 have been previously validated for use in Singapore [19].

### Knee extensor strength

A hand-held dynamometer (Lafayette, IN, USA) was used to measure isometric knee extensor strength based on the testing procedures described by Martin et al. [20]. A total of five raters – two physiotherapists and three technicians – obtained the knee strength (and ROM, *vide infra*) measurements. A “make” test was used as it was deemed more reliable than a “break” test [21]. Knee extensor strength was measured with the patients in supine position, and the knee was positioned in approximately 30^°^ flexion by a firm wedge. The dynamometer force pad was placed just proximal to the ankle joint and knee extensor strength was quantified in kilogram force. All patients performed two maximal trials for 3 to 5 seconds with a 30-second rest interval. Additional measurements were taken if the patient reported a failure to achieve maximum effort. The higher of two valid trials was recorded and normalized as a direct percentage of body weight (%BW) because we believe that this index of knee strength made intuitive sense to our patients. Although we [22] and others [23] have shown, using allometric analyses, that the association between muscle force generation and body weight is not a strict 1:1 ratio, analyzing our data using allometrically-scaled knee extensor strength did not qualitatively alter our study conclusions (data not shown). Also of interest, we performed strength testing in the supine position – and not in the seated position – with the intent to reduce the influence of the rater’s strength on the patient’s measurements [20]. We attempted to ensure that the same rater evaluated the same patient at each time point although this was not fully achievable due to the large patient volume in our hospital (~1,500 TKAs per annum [24]). Nevertheless, all technicians were trained and audited by authors HCC and WY who have a combined experience of over 30 years in orthopaedic and sports physiotherapy. Furthermore, for the supine knee strength measurements, previous studies have demonstrated good test–retest reliability (intraclass correlation coefficients, 0.80 to 0.86) and concurrent validity with Biodex dynamometry measurements [20,25].

### Knee extension ROM

A large standard goniometer (Jamar, Clifton, NJ, USA) was used to measure passive knee extension ROM. Knee extension ROM was measured with the patients in supine position with the heel elevated on a firm wedge. The axis of the goniometer was placed on the femoral lateral epicondyle. The proximal arm of the goniometer was directed toward the greater trochanter of the femur whilst the distal arm of the goniometer was directed toward the lateral malleolus of the ankle. Patients were asked to relax to allow the knee to passively extend. Two sets of measurements were taken, and the lower measurement (greater knee extension) was recorded. For the knee extension ROM measurements, one previous study in patients with knee OA has demonstrated good test–retest reliability (intraclass correlation coefficient = 0.85) [26].

### Statistical analysis

We used descriptive statistics to characterize the study sample: we used means (SDs) for continuous variables and percentages for categorical variables. Analysis of variance (ANOVA) for repeated measures was used to test for change over time in the SF-36 and knee measures. Pearson correlation was used to quantify the correlation between knee measures.

**Figure 1:**
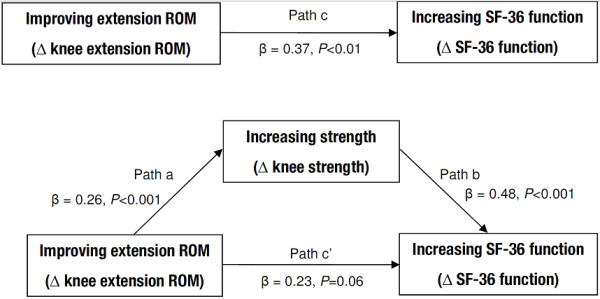
**Changes (pre to post TKA) in knee extensor strength mediated the association between changes in knee extension ROM and changes in SF-36 physical function.** For presentation clarity, covariates – namely, age, sex, body mass index, number of comorbidities, and changes in SF-36 bodily pain and mental health scores – are not displayed.

We used a mediation model to examine whether knee extensor strength mediated the influence of knee extension ROM on SF-36 physical function at the change scores (pre- to post TKA) level (Figure 1). Covariates in each model included age, sex, BMI, the number of comorbidities, and changes in SF-36 bodily pain and mental health scores. In the mediation model, the total effect (path c) of the independent variable (changes in knee extension ROM) on the dependent variable (changes in SF-36 physical function) comprises a direct effect (path c’) of the independent variable on the dependent variable and an indirect (mediation) effect of the independent variable on the dependent variable through the mediator (changes in knee extensor strength). The mediation effect through the mediator is quantified by the product of the regression coefficient of knee extension ROM on knee extensor strength (path a) and the regression coefficient of knee extensor strength on SF-36 physical function (path b). We used the INDIRECT macro [27] to estimate the mediation effect (path axb) and its bootstrapped (1,000 samples), bias-corrected and accelerated 95% confidence interval (95% CI). Mediation effects are considered statistically significant when zero is not contained within the 95%CIs [27]. Finally, in sensitivity analysis, we tested a reversed mediation model – wherein changes in knee extension ROM were due to changes in knee extensor strength – to assess the direction of the proposed mediation effects. All statistical analyses were done with PASW software, version 18, and R software, version 2.15.0.

## Results

### Sample characteristics

**Figure 2:**
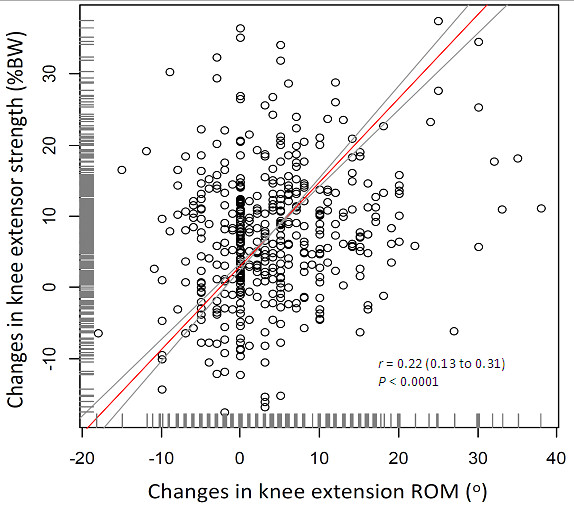
**Scattergram of changes in knee extensor strength versus changes in knee extension ROM in 441 patients.** The straight line represents ordinary least product regression line with its 95% confidence limits (curved lines): (changes in knee extensor strength) = 3.0 ( 2.5 to 3.4 ) + 1.18(1.10 to 1.29) X (changes in knee extension ROM). Observed change scores of the knee ROM and strength measures are indicated by the short vertical lines above the x- and y-axes, respectively.

Table 1 summarizes the participants’ descriptive characteristics, while Table 2 provides the descriptive statistics for the SF-36 physical function and knee measures. The patients were predominantly female (82%) and were on average moderately overweight (mean (SD) BMI, 28(4.8) kg/m^2^). All SF-36 and knee measures increased significantly 6 months following a TKA. Specifically, mean SF-36 physical function scores were 39(SD = 23) points preoperatively and 67(SD = 18) points 6 months postoperatively. With respect to the knee measures, knee extensor strength increased from preoperative level by around 40% at the 6 month postoperative assessment (18%BW vs. 26%BW; *P* < 0.001). Knee extension ROM improved significantly (*P* < 0.001) from 8.2° (knee flexion contracture) preoperatively to 4.3° postoperatively. Improvement in knee extension ROM was positively associated with increased knee extensor strength (*r* = 0.22, *P* < 0.001, Figure 2). Increased SF-36 physical function was positively associated with improvement in knee extension ROM (*r* = 0.15, *P* < 0.001) and with increased knee strength (*r* = 0.27, *P* < 0.001) (scattergrams are shown in Additional file 1).

**Table 1:** **Demographics and patient characteristics (*n*** **= 441)**

**Characteristic**	**Value**
Age, years	67.9 ± 7.8
Male sex, no. (%)	83 (19%)
Singaporean Chinese, no. (%)	389 (88%)
Height, m	1.54 ± 0.08
Body mass, kg	65.9 ± 13.1
BMI, kg/m^2^	27.9 ± 4.8
Overweight or obese, no. (%)	375 (85%)
Education, no. (%)	
Primary or less	335 (76%)
Secondary	84 (19%)
Tertiary	22 (5%)
Comorbidities, no. (%)	
None	66 (15%)
1 comorbidity	141 (32%)
2 comorbidities	137 (31%)
3 comorbidities	69 (16%)
≥4 comorbidities	28 (6%)

Note: Values reported were mean ± SD unless stated otherwise.

Abbreviations: *BMI*, body mass index.

**Table 2:** **Postoperative recovery of SF-36 and knee measures (*n*** **= 441)**

**Variables**	**Pre TKA**	**Post TKA**	***P*-value**^**†**^
SF-36 variables			
Physical function	38.8 (22.7)	66.6 (17.9)	<0.001
Bodily Pain	36.8 (18.3)	69.0 (23.5)	<0.001
Mental Health	75.9 (19.1)	82.7 (14.5)	<0.001
Knee variables			
Extensor strength, %BW	18.0 (8.0)	25.6 (8.6)	<0.001
Extension ROM, deg	8.2 (8.2)	4.3 (4.9)	<0.001

Note: Values reported were mean (SD) unless stated otherwise.

Abbreviations: SF-36 = Medical Outcomes Study 36-Item Short-Form Health Survey; *ROM*, range of motion; *BW*, body weight; *TKA*, total knee arthroplasty.

^†^*P*-values derived from repeated-measure ANOVA.

### Mediation analyses

Figure 1 shows the results of the mediational analyses. Overall, the mediator model accounted for ~29% of the variance in physical function (*P* < 0.001). Improving knee extension ROM was significantly associated with increasing physical function (*P* < 0.01 for path c, Figure 1). Controlling for covariates, the mediation effect of knee extensor strength on the association between changes in knee extension ROM and physical function was statistically significant (path axb = 0.13; 95% CI, 0.05 to 0.23). In contrast, no mediation effect was observed in the reversed mediation model (path axb = 0.04; 95% CI, -0.01 to 0.11).

## Discussion

The purpose of this study was to explore, in patients before and 6-months following a TKA, whether knee extension ROM had an indirect effect on SF-36 physical function through the putative pathway of knee extensor strength. Our results suggest that changes (pre to post TKA) in knee extensor strength mediated the association between changes in knee extension ROM and self-report physical function. To our knowledge, these findings have not been previously described in patients with TKA.

Our results, without considering mediating effects, are consistent with cross-sectional studies [7,9,12] of a positive association between knee extension ROM and physical function in TKA (path c in Figure 1). Furthermore, case series [28,29] and small trials [30] are available demonstrating that in patients before and following a TKA, rehabilitation interventions – for example, such as manual therapy and splinting – increased knee ROM and improved physical function. Overall, our large study using longitudinal change scores extends the previous literature to suggest that knee extension ROM is an important correlate of physical function in TKA.

Consistent with theoretical expectations, changes in knee extension ROM were associated with changes in knee extensor strength (Figure 2) which, in turn, were associated with changes in physical function (Figure 1). Furthermore, the results from the sensitivity analysis using a reversed mediation model support the direction of the proposed mediation. How do we explain our results? As mentioned in the Introduction, a knee flexion contracture (knee extension ROM > 0°) may potentially compress the force-length relationship [14,15] of the knee extensors such that strength production diminishes at lesser degrees (~30°) of knee flexion. As a corollary, our results indicate that deficits in knee extension ROM are a putative multivariate cause of knee extensor weakness in TKA. And if future studies can substantiate this notion, the discussion in the literature about whether knee ROM or knee muscle strength should be given greater prominence in TKA rehabilitation [31-33] would become moot.

Our study has limitations. First, although our use of change scores permitted a more rigorous exploration of mediation than do cross-sectional data, our study variables were measured concurrently at each time point which precluded an examination of temporal associations between variables [34]. Accordingly, to better assess the criterion of temporality, future longitudinal studies should evaluate the changes in the independent variable, mediator, and outcomes sequentially over different time points. Second, and relatedly, because we did not have multiple sequential measurements at successive time intervals, as pointed out by the reviewer, we were unable to evaluate whether the rate of change in knee extension ROM influenced (moderated) the proposed mediation effects. Third, we did not have any follow-up strength measurements later than 6 months post TKA; hence, we were unable to evaluate the effects of improving knee extension ROM post 6 months on knee extensor strength and functional ability. Finally, whilst we used a self-report measure of physical function to facilitate data collection, we acknowledge that performance-based measures of physical function are necessary because they provide important, complementary information about functional status [35].

## Conclusions

Our results indicate that knee extension ROM is an important correlate of physical function in patients with TKA. More important, changes in knee extension ROM and knee extensor strength interrelated to influence physical function. If future studies could demonstrate that the etiology of knee extensor muscle weakness is associated, *inter alia*, with knee extension ROM deficits, these findings would suggest that interventions to improve the range of knee extension may be useful in improving knee extensor performance.

## Abbreviations

OA: Osteoarthritis; ROM: Range of motion; TKA: Total knee arthroplasty; SF-36: Short form 36.

## Competing interests

The authors declare that they have no competing interests.

## Authors’ contributions

Authors YHP, PHO, HCC, WY, CT, and LNN (1) have all contributed to conception and design of this study; (2) have been involved in drafting the manuscript and revising it critically for important intellectual content; and (3) have given final approval of this version to be published.

## Pre-publication history

The pre-publication history for this paper can be accessed here:

http://www.biomedcentral.com/1471-2474/14/33/prepub

## Supplementary Material

Additional file 1 **Figure S1.** Flowchart of participant recruitment. TKA = Total Knee Arthroplasty, HTO = High Tibial Osteotomy, UKA = Unicompartmental Knee Arthroplasty. **Figure S2.** Scattergram of changes in SF-36 physical function scores versus changes in knee extension ROM in 441 patients. The straight line represents ordinary least product regression line with its 95% confidence limits (curved lines): (changes in SF-36 physical function) = 15.5 (14.4 to 16.5)  + 2.9 (2.7 to 3.2) X (changes in knee extension ROM). Observed change scores of the knee and SF-36 measures are indicated by the short vertical lines above the x- and y-axes, respectively. **Figure S3.** Scattergram of changes in SF-36 physical function scores versus changes in knee extension strength in 441 patients. The straight line represents ordinary least product regression line with its 95% confidence limits (curved lines): (changes in SF-36 physical function) = 8.1 (6.3 to 9.7) + 2.5 (2.3 to 2.7) X (changes in knee extensor strength). Observed change scores of the knee and SF-36 measures are indicated by the short vertical lines above the x- and y-axes, respectively. Click here for file
