# Supplementary material for: Knee extension range of motion and self-report physical function in total knee arthroplasty: mediating effects of knee extensor strength
Source: BMC Musculoskelet Disord. 2013 Jan 18;14:33. doi: 10.1186/1471-2474-14-33 (PMC3567935; doi:10.1186/1471-2474-14-33)
Supplement: Additional file 1 — Figure S1. Flowchart of participant recruitment. TKA = Total Knee Arthroplasty, HTO = High Tibial Osteotomy, UKA = Unicompartmental Knee Arthroplasty. Figure S2. Scattergram of changes in SF-36 physical function scores versus changes in knee extension ROM in 441 patients. The straight line represents ordinary least product regression line with its 95% confidence limits (curved lines): (changes in SF-36 physical function) = 15.5 (14.4 to 16.5) + 2.9 (2.7 to 3.2) X (changes in knee extension ROM). Observed change scores of the knee and SF-36 measures are indicated by the short vertical lines above the x- and y-axes, respectively. Figure S3. Scattergram of changes in SF-36 physical function scores versus changes in knee extension strength in 441 patients. The straight line represents ordinary least product regression line with its 95% confidence limits (curved lines): (changes in SF-36 physical function) = 8.1 (6.3 to 9.7) + 2.5 (2.3 to 2.7) X (changes in knee extensor strength). Observed change scores of the knee and SF-36 measures are indicated by the short vertical lines above the x- and y-axes, respectively. [file 1471-2474-14-33-S1.doc]

**Pre-operative assessment**

Consecutive patients operated by

three high-volume surgeons between January 2006 and January 2009

(n = 836)

**Exclusions** (n=395)

152 Contralateral TKA within 12 months

before and 6 months after index TKA

30 History of stroke of other

neurological conditions

12 History of lower limb fracture

26 Previous hip arthroplasty or HTO

4 Previous UKA on index knee

64 Developed complications prior to follow-up session

48 Missing knee data

59 Preoperative knee hyperextension

**6 months post-operative assessment**

Patients included in analysis (n=441)

**Supplementary Figure 1**.

Flowchart of participant recruitment. TKA = Total Knee Arthroplasty, HTO= High Tibial Osteotomy, UKA= Unicompartmental Knee Arthroplasty

| *r* = 0.15 (0.06 to 0.24)  *P* < 0.01 |
| --- |
| **Supplementary Figure 2.**  Scattergram of changes in SF-36 physical function scores versus changes in knee extension ROM in 441 patients. The straight line represents ordinary least product regression line with its 95% confidence limits (curved lines): (changes in SF-36 physical function) = 15.5(14.4 to 16.5) + 2.9(2.7 to 3.2) X (changes in knee extension ROM). Observed change scores of the knee and SF-36 measures are indicated by the short vertical lines above the x- and y-axes, respectively. |

| *r* = 0.27 (0.18 to 0.36)  *P* < 0.0001 |
| --- |
| **Supplementary Figure 3.**  Scattergram of changes in SF-36 physical function scores versus changes in knee extension strength in 441 patients. The straight line represents ordinary least product regression line with its 95% confidence limits (curved lines): (changes in SF-36 physical function) = 8.1(6.3 to 9.7) + 2.5(2.3 to 2.7) X (changes in knee extensor strength). Observed change scores of the knee and SF-36 measures are indicated by the short vertical lines above the x- and y-axes, respectively. |
